# P62 Regulates resveratrol-mediated Fas/Cav-1 complex formation and transition from autophagy to apoptosis

**DOI:** 10.18632/oncotarget.2733

**Published:** 2014-11-29

**Authors:** Jun Zhang, Ke Ma, Tingting Qi, Xiaoning Wei, Qing Zhang, Guanwu Li, Jen-Fu Chiu

**Affiliations:** ^1^ Open Laboratory for Tumor Molecular Biology/Department of Biochemistry, The Key Lab of Molecular Biology for High Cancer Incidence Coastal Chaoshan Area, Shantou University Medical College, Shantou, China

**Keywords:** apoptosis, autophagy, cell death, resveratrol, Fas/Cav-1 complex, P62

## Abstract

Resveratrol is a potential polyphenol drug used in cancer treatment. We examined the relationship between autophagy and apoptosis in RSV-treated non-small lung adenocarcinoma A549 cells. Resveratrol treatment increased autophagy and autophagy-mediated degradation of P62. Immunocytochemistry revealed P62 co-localized with Fas/Cav-1 complexes, known to induce apoptosis. However, siRNA-mediated P62 downregulation enhanced formation of Fas/Cav-1 complexes, suggesting that P62 inhibited Fas/Cav-1 complex formation. Fas/Cav-1 complexes triggered caspase-8 activation and cleavage of Beclin-1, releasing a C-terminal Beclin-1 peptide that translocated to the mitochondria and initiate apoptosis. Inhibition of autophagy by siRNA-mediated repression of Beclin-1 also blocked RSV-induced apoptosis, showing a dependence of apoptosis on autophagy. P62 knockdown by siRNA accelerated the activation of caspase-8 and initiate apoptosis, while Cav-1 knockdown inhibited apoptosis, but increased autophagy. Inhibition of autophagy by 3-MA prevented both P62 degradation and induction of apoptosis, whereas inhibition of apoptosis by z-IETD-FMK or z-DEVD-FMK enhanced both P62 induction and autophagic cell death. In conclusion, P62 links resveratrol-induced autophagy to apoptosis. P62 blocks apoptosis by inhibiting Fas/Cav-1 complex formation, but RSV-induced autophagic degradation of P62 enables formation of Fas/Cav-1 complexes which then activate caspase-8-mediated Beclin-1 cleavage, resulting in translocation of the Beclin-1 C-terminal fragment to the mitochondria to initiate apoptosis.

## INTRODUCTION

Resveratrol (trans 3, 4′, 5-trihydroxystilbene; RSV), a polyphenol phytoalexin found in grapes, peanuts and other plants, is well-known for its potential antioxidant, anti-tumorigenic activities [1–4], extends lifespan by activate Sirt1 [5, 6]. Accumulated reports demonstrate that RSV has the ability to affect tumor initiation and promotion, arrest angiogenesis and metastasis, and induce cell cycle arrest and apoptosis [7]. Previous studies have indicated that resveratrol inhibits the proliferation of A549 non-small adenocarcinoma cells and induces apoptosis [8]. Studies on autophagy induced by resveratrol in A549 cells have also attracted an immense amount of attention for its potential antitumor activity [9]. Additionally, RSV is currently in phase I clinical development with preliminary evidence of antitumor activity [10]. However, the actual mechanisms of RSV-induced cell death and the specific molecular targets of RSV in A549 cancer cells have not yet fully established.

Autophagy, literally interpreted as “self-eating”, is a highly evolutionarily conserved catabolic process in eukaryotes and plays an important role in the recycling of cellular components [11]. It is involved in various biological processes, such as adaptation to changing environmental conditions, cellular remodeling during development and differentiation, and cancer [12]. Crosstalk between apoptosis and autophagy exists to regulate cell death. Recent studies have shown that several molecules required for autophagy also play a key role in apoptosis. For example, cleavage of Beclin-1, a well-known protein required for the initiation of the formation of the autophagosome, generates a pro-apoptotic protein fragment that translocates to the mitochondria and interacts with the antiapoptotic Bcl-2 family protein Bcl-xL to antagonize autophagy and initiate mitochondrial apoptosis [13, 14]. An increasing number of reports show that members of the caspase family, such as caspase-3 and caspase-8, participate in the cleavage of Beclin-1 [15, 16].

The apoptosis initiator Fas, is a well-documented mediator in the apoptotic process. Activated Fas induces the cleavage and activation of caspase-8, which induces apoptosis through caspase-3 activation or cleaves t-Bid to change the mitochondrial membrane potential (MMP) [17, 18]. Caveolin-1 (Cav-1), as a scaffolding protein within the plasma membrane microdomains, is an important regulator that interacts between signaling proteins and Fas [19]. Recent emerging evidences suggest that Cav-1 plays a critical role in the regulation of a wide range of cellular processes, including the regulation of signal transduction, cell death, and survival [19]. However, it is still unknown the actual function of Cav-1 involved in autophagy.

The signaling adaptor P62 protein, also called sequestosome 1 (SQSTM1), is commonly found in inclusion bodies containing polyubiquitinated protein aggregates. P62 is a functional protein with multiple-binding domains that can combine with several proteins and bring them into the autophagolysosome for degradation during autophagy [20]. Recent work reveals that P62 acts as a signaling hub through its ability to recruit and oligomerize signaling molecules in cytosolic speckles to control cell survival and apoptosis, and elimination of P62 by autophagy suppresses tumorigenesis [21]. The overexpression of p62 contributed to ROS production in a positive feedback loop, thereby leading to increased genome instability [22]. Other reports showed that p62 was able to maintain and stabilize the integrity and functions of mitochondria for the longevity or immortalization of mammalian cells [23]. The accumulation of p62 reflected the inhibition of proteasomal activities [24]. These discoveries suggest that P62 is a central player in the life and death decisions of cells. The role of P62 involved in the linkage between autophagy and apoptosis is still not yet fully understood.

We and others have previously demonstrated that RSV drives tumor cells toward autophagic cell death [9]. In this study, we investigate whether p62 plays a role in regulating the processes of autophagy and apoptosis, and how p62 regulates the switch between these two processes. Here we demonstrate that in human A549 cells, cell death undergoes a transition from autophagy to apoptosis after prolonged treatment with RSV (more than 48 hours). The switch from autophagy to apoptosis is governed by P62-mediated formation of Cav-1 and Fas complexes that activate cleavage of caspase-8 and Beclin-1 to regulate autophagy and apoptosis.

## RESULTS

### RSV induces growth inhibition and cell death in A549 cells

**Figure 1:**
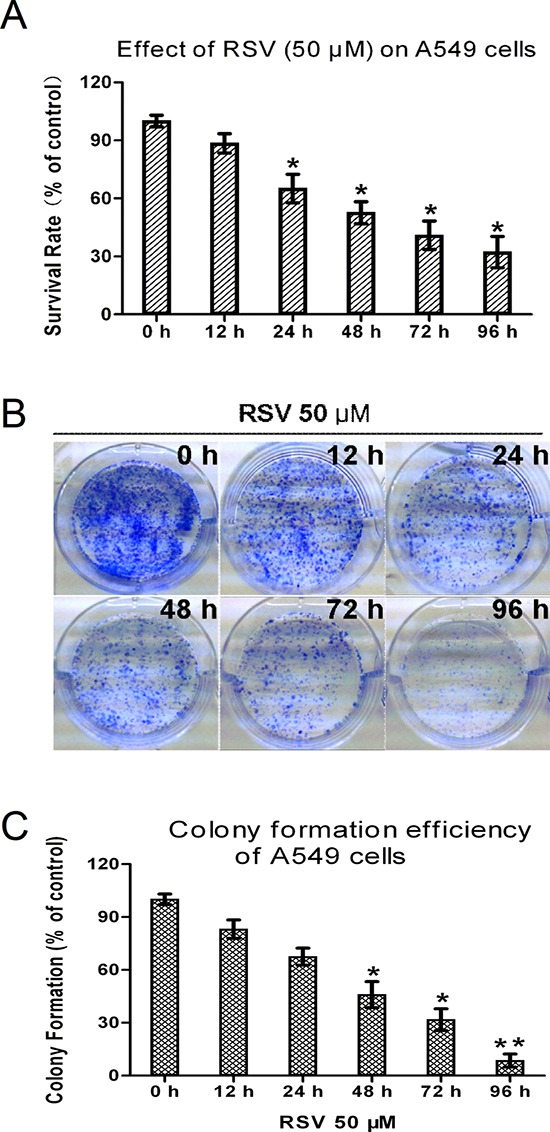
Effects of RSV on cell proliferation of A549 cells **(A)** Time-dependent effect of RSV on cell proliferation of A549 cells. Cells were plated on 96-well plates at 7 × 10^3^ cells/well overnight, then cultured with 50 μM RSV for the indicated times, and cell proliferation determined by MTT assay. **(B)** Photomicrographs show representative colony formation of A549 cells treated with 50 μM RSV at the indicated times. **(C)** Number of colonies after 50 μM RSV treatment for the indicated times. Columns indicate mean ± SD of three experiments, *p < 0.05 vs. respective control cells.

To investigate the cytotoxic activity of RSV on A549 cells, exponentially growing cells were incubated with 50 μM RSV, then cell viability was measured at various times following RSV addition (Fig. 1A). The number of viable cells decreased in a time-dependent manner to 65.8% and 40% at 24 and 72 h, respectively, after RSV treatment. Further analysis by clonogenic assay demonstrated that RSV treatment caused a reduction in both the size and number of colonies, in agreement with our cell proliferation assay (Fig. 1B & 1C). Thus, the data suggested that RSV arrested A549 cell proliferation and induced cell death.

### RSV induces early autophagy followed by apoptosis in A549 cells

**Figure 2:**
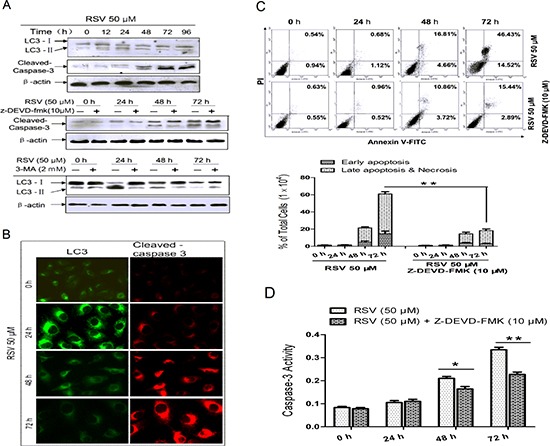
RSV-induced autophagy and apoptosis at different time points in A549 cells **(A)** A549 cells were treated with 50 μM RSV, in the presence or absence of 3-MA and Z-DEVD-FMK for the indicated time, then whole-cell lysates from control and RSV-treated cells were subjected to SDS-PAGE, and the levels of LC3 and caspase-3 were analyzed by immunoblotting. Actin was used as a loading control. **(B)** Autophagy (LC3) and apoptosis (cleaved caspase-3) were detected by immunofluorescence as described in the Materials and Methods. Cells were treated with 50 μM RSV for the indicated time and then stained with anti-LC3 and anti-cleaved caspase-3 antibodies. **(C)** A549 cells were pre-incubated with or without Z-DEVD-FMK, and then treated with 50 μM RSV for the indicated times before detection of cell death by flow cytometry after staining with FITC-conjugated Annexin-V and PI. The histogram represented quantification analysis based on three independent experiments. **(D)** Effect of RSV on the activity of caspase-3. Cells were treated with 50 μM RSV for the indicated times in the presence or absence of Z-DEVE-FMK. Data are means ± SD of three individual determinations, *p < 0.05 and **p < 0.01 vs. respective control cells.

To investigate RSV-induced autophagy and apoptosis in A549 cells, we exposed cells with 50 μM RSV for up to 96 h. Both autophagy and apoptosis were induced by RSV in a time-dependent manner, as indicated by markers LC3 and cleaved caspase-3. As shown in Fig. 2A, LC3II emerged after RSV treatment for 12 h, and reached a peak around 24 h, followed by appearance of cleaved caspase-3 at 48 h, which became more prominent with time. To test the case, cells were pre-treated with 3-methyladenine (3-MA) (Fig. 2A) or Z-Asp (OMe)-Glu (OMe)-Val-Asp (OMe)-FMK (Z-DEVD-FMK) (Fig. 2A) for 1 hour before RSV treatment and then analyzed for autophagy and apoptosis. We also analyzed apoptotic morphologic changes by DAPI staining (Supplementary Fig. 1A) and autophagic vacuole formation by staining with the autolysosome indicator monodansylcadaverine (MDC) (Supplementary Fig. 1B). The results demonstrated that RSV induced apoptosis 48 h after treatment with RSV, which was strongly reduced by Z-DEVD-FMK, whereas RSV-induced autophagy was evident by 12 h, increased up to 24 h, then dramatically dropped to low levels by 48 h.

To further confirm that autophagy and apoptosis are both activated in RSV-treated cells, we then detected LC3 and cleaved caspase-3 simultaneously by immunofluorescence staining in the same samples (Fig. 2B). Results indicated that the number of LC3 speckles increased prior to 48 h, and cleaved caspase-3 increased after 48 h in a time-dependent manner. Next, we determined apoptotic cells by flow cytometry (Fig. 2C). The non-treated cells, as well as cells treated with RSV for less than 48 h, had a low percentage of apoptotic cells (2% and less than 20%, respectively). However, when cells were incubated with RSV for 72 h, the percentage of apoptotic cells increased to 61%. This increase in apoptosis was blocked by the caspase-3 inhibitor, Z-DEVD-FMK (decreased to 18.4%). Enzymatic activity of caspase-3 also increased after 48 h following RSV treatment (Fig. 2D), which could be inhibited by incubation of cells with Z-DEVD-FMK. In conclusion, the above results indicate that autophagy and apoptosis are both activated by RSV in a specific time course, and induction of autophagy occurs prior to the activation of apoptosis.

### RSV activates caspase-8 and its functions in autophagy and apoptosis

**Figure 3:**
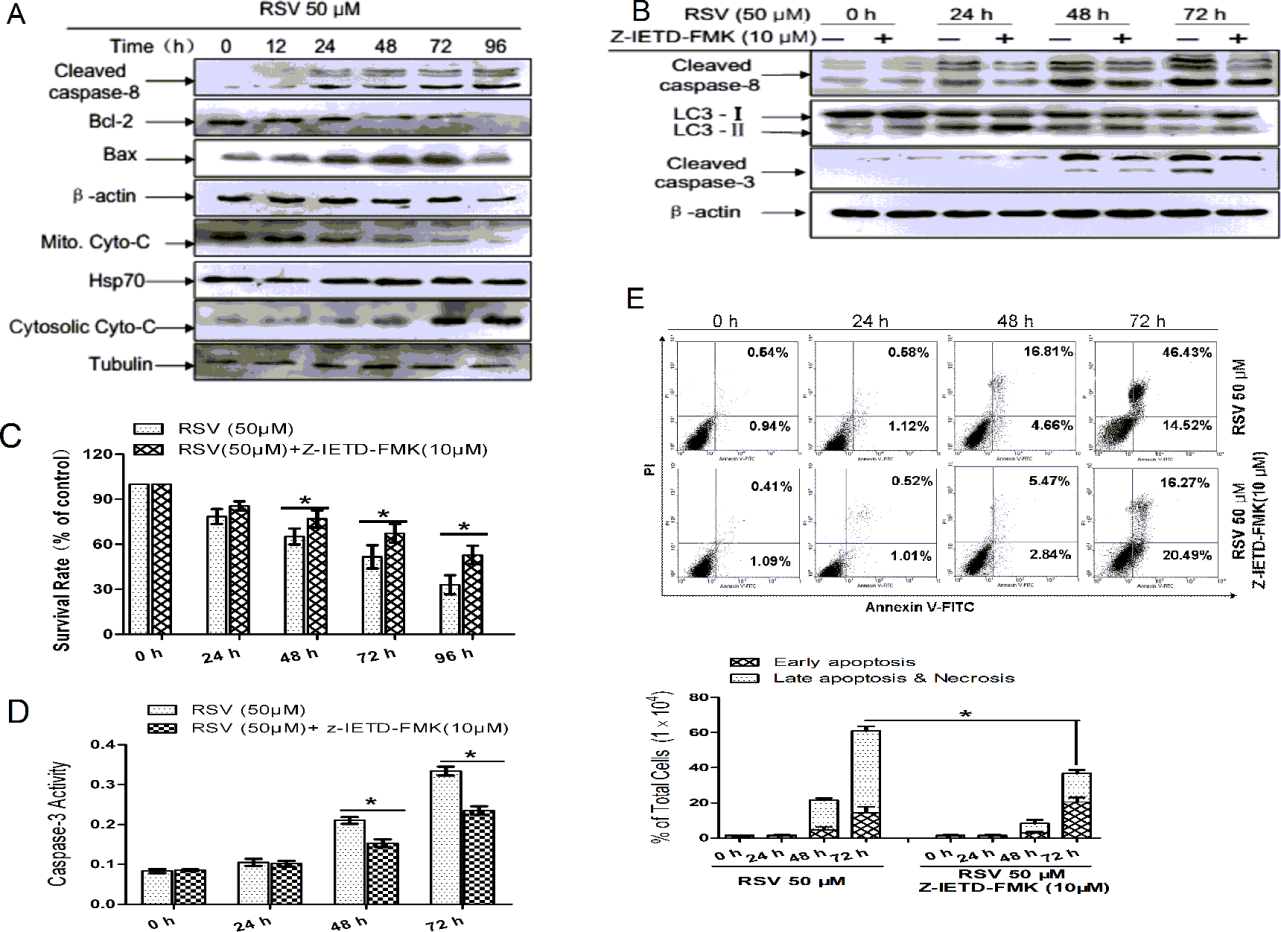
Caspase-8 in autophagy and apoptosis in RSV-treated A549 cells **(A)** Western blot analysis detected apoptosis-related protein expression levels after treatment with 50 μM RSV at the indicated times. **(B)** Cells were incubated with 50 μM RSV for the indicated times in the presence or absence of Z-IETD-FMK, then cell lysates were used to analyze the expression of cleaved caspase-8, cleaved caspase-3 and LC3 with the specific antibodies. β-actin was used as the loading control. **(C)** Cells were exposed to 50 μM RSV plus Z-IETD-FMK for the indicated times, cell viability was determined by MTT assay. **(D)** Cells were exposed to RSV combined with Z-IETD-FMK for the indicated times. After exposure, cell lysates were obtained and caspase-3 enzymatic activities were measured. **(E)** A549 cells were pre-incubated with or without Z-IETD-FMK and then treated with 50 μM RSV for the indicated times before detection of cell death by flow cytometry after the staining of FITC-conjugated Annexin-V and PI. The histogram represented quantification analysis based on three independent experiments. Data are means ± SD of three individual determinations, *p < 0.05 and **p < 0.01 vs. respective control cells.

To determine whether caspase-8 is involved in RSV-induced autophagy and apoptosis, we examined cleaved caspase-8 by Western blot. Caspase-8 cleavage forms were clearly detectable at 24 h after RSV treatment (Fig. 3A), and the ratio of Bax/Bcl-2 increased with time, especially after 48 h. This correlated well with the timing of cytochrome C release from the mitochondria to the cytosol (Fig. 3A). Combined treatment with Z-Ile-Glu(OMe)- Thr-Asp(OMe)-FMK(Z-IETD-FMK) could reverse RSV-induced apoptosis (Fig. 3B). To confirm that apoptosis was induced through a change in mitochondrial membrane permeability, we then analyzed the mitochondrial membrane potential (MMP) with the indicator Rho123. As shown in Supplementary Fig. 2A, the mean fluorescence intensity was reduced significantly at 48 h after treatment with RSV, illustrating that MMP was decreased by the action of RSV in a time-dependent manner, and caspase-8 inhibition could reduce the action.

Next, we examined cell viability by MTT assay. Cells in the presence of Z-IETD-FMK had a higher survival rate compared with cells in the absence of the caspase-8 inhibitor (Fig. 3C). We then detected the effect of Z-IETD-FMK on caspase-3 enzymatic activity (Fig. 3D). The results from flow cytometry showed that Z-IETD-FMK reduced the percentage of apoptotic cells (Fig. 3E). In conclusion, RSV activated caspase-8 and then triggered apoptosis.

To further demonstrate the role of caspase-8 in autophagy, we examined the levels of LC3-I and II in the prescence or absence of Z-IETD-FMK. Conversion of LC3-I to II was increased in the presence of the caspase-8 inhibitor (Fig. 3B), suggesting that caspase-8 may function as an inhibitor of autophagy. We next examined the co-expression of cleaved caspase-8 and the autophagy marker-LC3, following RSV addition, by immunofluorescence. The amount of LC3 co-expressed with cleaved caspase-8 significantly increased after RSV treatment for 24 h and became more evident in the combined treatment of RSV and Bafilomycin A, an inhibitor of autophagic protein degradation (Supplementary Fig. 2B), suggesting that caspase-8 could be degraded by autophagy. From the above results we conclude that activation of caspase-8 functions as a switch to inhibit autophagy and at the same time to initiate apoptosis. Therefore our results demonstrate that autophagy promotes the cleavage and activation of caspase-8.

### Caspase-8 cleaves Beclin-1 to inhibit RSV-induced autophagy and promote apoptosis

**Figure 4:**
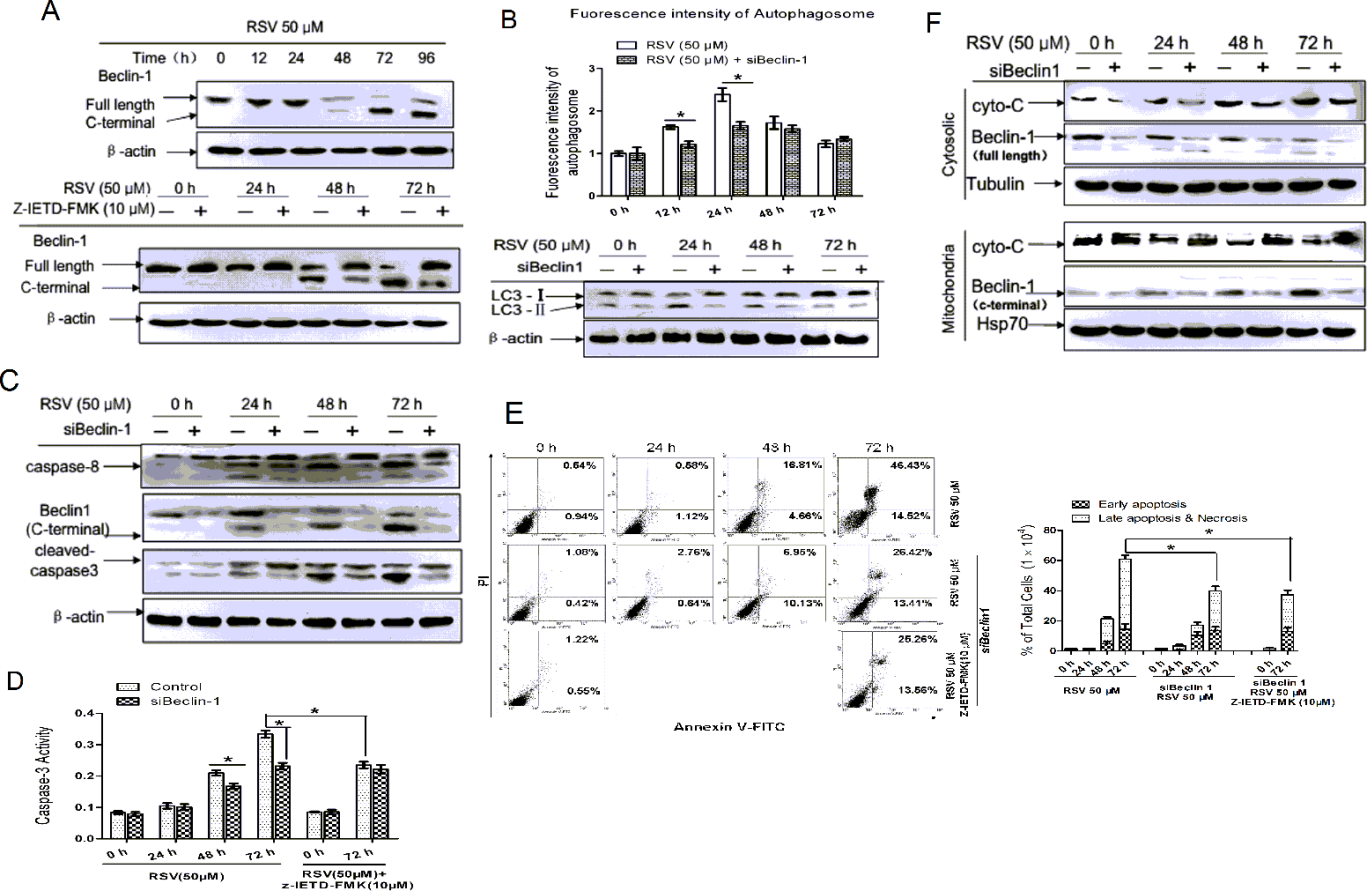
Caspase-8 cleaved Beclin-1 inhibits RSV-induced autophagy and promotes apoptosis **(A)** Beclin-1 was analyzed with full-length and C-terminal antibodies after cells were treated with 50 μM RSV for the indicated times in the presence or absence of Z-IETD-FMK. **(B)** Autophagy markers were detected by MDC staining (upper) and western blot (below). A549 cells were transfected with or without Beclin-1 siRNA and incubated with 50 μM RSV for the indicated times, then stained with MDC, or harvested to extract protein for SDS-PAGE analysis. **(C)** Cells were transfected with or without Beclin-1 siRNA and then incubated with 50 μM RSV for the indicated times. Proteins were then extracted and analyzed by western blot using the antibodies as indicated. **(D)** Effect of RSV on the activity of caspase-3 in wildtype and Beclin-1-deficient cells. Cells were treated with 50 μM RSV combined with or without Z-IETD-FMK for the indicated times. **(E)** Cells were treated with 50 μM RSV combined with or without Z-IETD-FMK in wildtype and Beclin-1-deficient cells for the indicated times. Apoptotic cells were analyzed by flow cytometry after staining with FITC-conjugated Annexin-V and PI. The histogram represented quantification analysis based on three independent experiments. **(F)** Cells were transfected with Beclin-1 siRNA and incubated with RSV, then harvested and separated into cytosolic and mitochondria-enriched fractions. Fractions were extracted and lysates were analyzed by western blot using antibodies against Beclin-1 and cyto-C. All data are the mean value of at least 3 independent experiments. *p < 0.05 compared with the control cells.

Because caspase-8 can cleave Beclin-1 to release a pro-apoptotic peptide, we defined the role of Beclin-1 in autophagy and apoptosis by first examining the expression of Beclin-1 mRNA by Q-PCR. Expression of Beclin-1 increased in a time-dependent manner following RSV addition (Supplementary Fig. 3A). We next detected the expression of full length Beclin-1and Beclin-1 cleavage products by western blot. Interestingly, cleaved Beclin-1 started to appear at 48 h and continued to increase up to 96 h. Conversely, the level of full length Beclin-1 gradually declined (Fig. 4A upper panel). Therefore, we conclude that RSV activates cleavage of Beclin-1, and this action requires caspase-8 (Fig. 4A lower panel).

The functional consequences of Beclin-1 in autophagy and apoptosis were examined using Beclin-1 siRNA knockdown. Results shown in Fig. 4B indicated that MDC fluorescence was dramatically decreased in Beclin-1 siRNA-transfected cells, and the conversion of LC3-I to LC3-II was reduced. These results clearly showed that Beclin-1 deficiency results in inhibition of autophagy. Intriguingly, we found that when Beclin-1 was knocked down, cell conversion to apoptosis was inhibited (Fig. 4C). Enzymatic activity of caspase-3 (Fig. 4D) and flow cytometry (Fig.4E) indicated that the extent of apoptosis in Beclin-1-deficient cells was reduced. MTT assay indicated that cell viability was substantially increased in Beclin-1 knockdown cells following RSV addition, and caspase-8 inhibition had little effect on cell survival, although caspase-3 inhibition increased survival (Supplementary Fig. 3B). Therefore, the above results suggested that Beclin-1 cleavage by caspase-8 is required for RSV-mediated apoptosis.

To confirm the truncated form of Beclin-1 indeed functions to enhance apoptosis, we examined the localization of Beclin-1 in mitochondria. Cleaved Beclin-1 was found to translocate to the mitochondria in a time-dependent manner, but full length Beclin-1 was mainly localized in the cytosol. Translocation was dramatically inhibited by Z-IETD-FMK (Supplementary Fig. 3C). As shown in the figure, the green color represents mitochondrial, the red color represents FL-Beclin-1 and C-Beclin-1, C-Beclin-1 aggregates and form big dots, which co-localized with green perfectly, but the full-Beclin-1 in the figure diffused outside the nuclear. To further analyze the function of cleaved Beclin-1 in apoptosis, we extracted the mitochondrial and cytosolic subcellular fractions to determine the levels of Beclin-1 by western blot (Fig. 4F). Beclin-1 localized in the mitochondria was mainly in its cleaved form, whereas full length beclin-1 was present in cytosol. In addition, RSV-induced cytochrome C release from the mitochondria to cytosol was decreased following Beclin-1 knockdown. In sum, the results above suggest that the cleavage of Beclin-1 by caspase-8 could inactivate autophagy and subsequently induce cell death through the intrinsic mitochondrial apoptotic pathway.

### Degradation of P62 by autophagy regulates RSV-induced apoptosis through Beclin-1 cleavage

**Figure 5:**
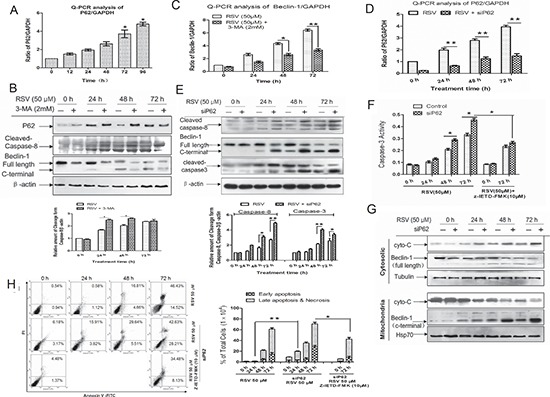
Degradation of P62 by autophagy regulates RSV-induced apoptosis through Beclin-1 cleavage **(A)** P62 mRNA was analyzed after 50 μM RSV treatment for the indicated times by quantitative real-time PCR. **(B)** Effect of 3-MA on p62 protein level, as well as cleaved caspase-8 and Beclin-1 were detected by western blot. Cells were treated with RSV or combined with 3-MA for the indicated times, then cell lysates were collected and subjected to immunoblotting. **(C)** Effect of 3-MA on Beclin-1 mRNA level was analyzed by real-time PCR. Cells were treated with 50 μM RSV, RSV + 3-MA (2 mM) for the indicated times. Cells were transfected with or without P62 siRNA and then treated with 50 μM RSV for the indicated times, and then **(D)** the knockdown efficiency was analyzed by real-time PCR, **(E)** Cell lysates were analyzed by immunoblotting using antibodies as indicated. The histogram represented quantification analysis based on three independent experiments. **(F)** Cells were transfected with P62 siRNA and then exposed to RSV in the presence or absence of Z-IETD-FMK for the indicated times. After exposure, cell lysates were obtained and caspase-3 enzymatic activities were measured. **(G)** Cells were transfected with P62 siRNA and incubated with RSV, then harvested and separated into cytosolic and mitochondrial fractions. Fractions were extracted and extracts were analyzed by western blot using Beclin-1 and cyto-C antibodies. **(H)** Cells were treated with 50 μM RSV combined with or without Z-IETD-FMK in wild-type and P62-deficient cells for the indicated times, then apoptotic cells were analyzed by flow cytometry after the staining of Annexin-V and PI. The histogram represented quantification analysis based on three independent experiments. Columns indicate mean ± SD of three experiments, *p < 0.05 vs. respective control cells.

To investigate the involvement of P62 in autophagy and apoptosis, we analyzed P62 mRNA after treatment with RSV by real-time PCR (Fig. 5A). P62 expression rose in a time-dependent manner. Combined treatment with 3-MA and RSV increased the level of p62 protein and the cleavage form of Caspase-8 (was also observed in Supplementary Fig. 2B) compared with the group treated with RSV alone (Fig. 5B). However, one intriguing observation is that 3-MA decreased Beclin-1 levels, but not through inhibition of cleavage since the cleavage form of Beclin-1 also reduced in 3-MA combined treatment group (Fig. 5B). To determine whether the reduction of Beclin-1 protein by 3-MA was caused by inhibiting gene transcription, we examined the level of Beclin-1 mRNA after treatment with 3-MA (Fig. 5C). 3-MA treatment markedly lowered the level of Beclin-1 mRNA, with a temporal pattern consistent with the change in protein level.

To further investigate the function of P62, we then knockdown P62 by using P62 specific siRNA, the knockdown efficiency was shown in Fig. 5D. Our results showed that P62 knockdown augmented cell apoptosis (Fig. 5E & 5F). To further elaborate the involvement of P62 in Beclin-1-mediated apoptosis, we determined the levels of full-length and cleaved Beclin-1 in the mitochondrial and cytosolic fractions by western blot analysis. Immunoblotting indicated RSV induced cleavage and mitochondrial translocation of Beclin-1 following p62 knockdown (Fig. 5G). In addition, the release of cytochrome C from the mitochondria to cytosol was enhanced in P62-deficient cells. (Fig. 5G). Results in Supplementary Fig. 4A further indicated that the Beclin-1 cleavage fragment was localized in mitochondrial compartment, and full length Beclin-1 was localized in cytosol in P62 knockdown cells.

To further confirm the involvement of P62 in apoptosis, we analyzed the apoptosis-related proteins by western blotting (Supplementary Fig. 4B). The data showed that P62-deficient cells had a higher extent of apoptosis, which was blocked by caspase-8 inhibition. These results were also confirmed by flow cytometry (Fig. 5H). MTT assay demonstrated that wild-type A549 cells had a higher survival ratio than cells treated with P62 siRNA (Supplementary Fig. 4C). All the above showed clearly that caspase inhibitors could block apoptosis induced by knocking down P62. In conclusion, when P62 was degraded by autophagy, the low level of P62 resulted in activation of caspase-8 to induce the cleavage of Beclin-1, whose cleavage product then functioned as an effector to initiate apoptosis through the mitochondrial apoptotic pathway.

### Fas/Cav-1 complex links P62 and Beclin-1 to transition from autophagy to apoptosis

**Figure 6:**
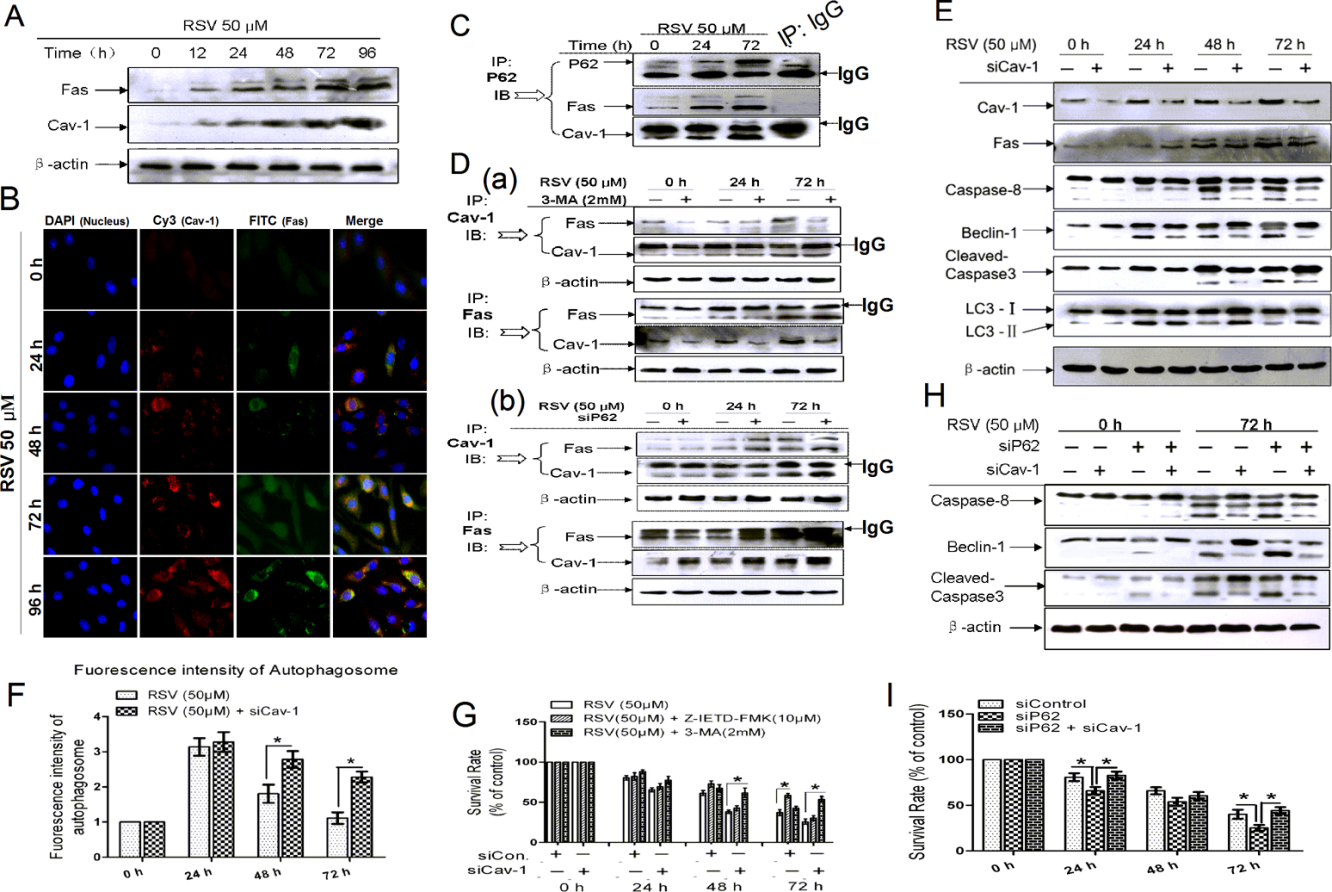
Fas/Cav-1 complex formation functions as a key regulator for P62-mediated apoptosis **(A)** A549 cells were treated with 50 μM RSV for up to 96 h, then cells were harvested and cell lysates were analyzed by immunoblotting. **(B)** Co-localization of Fas and Cav-1 following RSV treatment, showing the interaction and expression level of the two proteins. Cells were stained with anti-Fas, anti-Cav-1, and DAPI, and then captured by microscopy after treatment with 50 μM RSV for the indicated times. **(C)** Fas/Cav-1 co-immunoprecipitated following RSV treatment. Cells were exposed to 50 μM RSV for the indicated times, then harvested, and cell lysates were used in the co-IP assays with P62 antibody. Immunoprecipitates were analyzed with anti-P62, anti-Fas and anti-Cav-1 antibodies. The purified IgG group (72 h) used as control to the p62 antibody we used. **(D)** A549 cells were pre-treated with 3-MA (a) or transfected with P62 siRNA (b), then exposed to 50 μM RSV for the indicated times. Cells were lysed and lysates were used in the co-IP assays with Fas or Cav-1 antibodies. The interaction between Fas and Cav-1 was determined using the corresponding antibody. **(E)** Cells were transfected with or without Cav-1 siRNA and then incubated with 50 μM RSV for the indicated times, then proteins were extracted and analyzed by western blot. **(F)** The viability of wild type and Cav-1 knockdown cells was analyzed by MTT assay. Cells were treated with 50 μM RSV in the presence or absence of autophagy and caspase-8 inhibitors. **(G)** Cells were co-transfected with Cav-1 and P62 siRNA, incubated with 50 μM RSV for indicated times, then extracted and analyzed by western blot. **(H)** Viability of wild type and P62, P62 and Cav-1 knockdown cells was analyzed by MTT assay after cell treatment with 50 μM RSV for 0 to 72 h. Columns indicate mean ± SD of three experiments, *p < 0.05 vs. respective control cells.

Cav-1 is a plasma membrane-localized scaffolding protein that links Fas to down-stream signaling pathways [19, 25]. The involvement of Cav-1 and Fas in RSV-induced cell apoptosis is still unknown. To increase our understanding of the conversion from autophagy to apoptosis by P62 and Beclin-1, we analyzed the co-localization of Fas with Cav-1. Both Fas and Cav-1 increased in expression and enhanced their co-localization after exposure to RSV (Fig. 6A & 6B). We then examined the role of the Fas/Cav-1 complex in the conversion from autophagy to apoptosis. P62 co-localized with Fas and Cav-1 in a time-dependent manner from 24 h to 72 h (Fig. 6C), the related purified-IgG pull down group loaded as control to the specific antibody we used. Treatment with 3-MA induced aggregation of P62 (as shown in Fig. 5B) and decreased the amount of Fas-Cav-1 interaction (Fig. 6D-a). However, P62 siRNA-transfected cells displayed higher Fas-Cav-1 interaction compared with wild type cells, regardless of whether Fas or Cav-1 antibody was used to detect the interaction complex (Fig. 6D-b), but there was no change in total levels of Fas and Cav-1 in cells treated or not treated with 3-MA and P62 siRNA. These results suggest that P62 regulates RSV-induced apoptosis by mediating the Fas and Cav-1 complex formation.

We next investigated whether Cav-1 affects the collective changes in caspase-8, Beclin-1, caspase-3 and apoptotic proteins. Cav-1 knockdown inhibited RSV-induced cleavage of caspase-8, Beclin-1, and caspase-3, (Fig. 6E), suggesting that Cav-1 deficiency suppresses apoptosis. Cav-1 knockdown alone did not change cell viability, as judged by MTT assay (Fig. 6G). However, 3-MA inhibited cell death of RSV-treated Cav-1-deficient cells, but Z-IETD-FMK had no effect. These results suggest that knockdown of Cav-1 blocked the activation of apoptosis within cells that were still undergoing autophagy. To confirm this, we analyzed LC3 by western blot. As shown in Fig. 6E, conversion of LC3-I to LC3-II was enhanced in Cav-1-deficient cells. We then stained for autophagosomes with MDC. MDC fluorescence was increased in Cav-1-deficient cells (Fig. 6F). We therefore concluded that Cav-1 is required for conversion from autophagy to apoptosis.

To expand our knowledge regarding the role of Cav-1 in apoptosis in P62-deficient cells, we co-transfected P62 siRNA and Cav-1 siRNA in A549 cells. Cav-1 knockdown inhibited the activation of caspase-8 and caspase-3, as well as cleavage of Beclin-1 compared with cells transfected with P62 siRNA alone (Fig. 6H). Moreover, Cav-1 knockdown could reverse the cell death induced by P62 knockdown (Fig. 6I). In summary, the above results suggest that Fas and Cav-1 complex formation is important in RSV-induced apoptosis, and Cav-1 functions to protect cells by blocking the transition from autophagy to apoptosis by forming a complex with Fas.

## DISCUSSION

The effect of RSV on cancer cells has mainly focused on antiproliferative and anticarcinogenic effects through cell cycle arrest, induction of apoptosis and sensitization to chemotherapy- induced apoptosis [25]. Recently, more and more research demonstrates that RSV also induces autophagy in different cells [9, 25]. However, the actual signaling pathways mediated by RSV to induce cell death remain elusive. Our previous study found that RSV induced autophagy in A549 cells through the Ca^2+^-AMPK-mTOR pathway [9]. Recently, autophagy and apoptosis, and the relationship between them are a subject of intense debate. Successive activation of both autophagy and apoptosis are observed in many systems. In this study, we observe degradation of P62 by autophagy after cell treatment with RSV, which results in time-dependent cell death associated with the activation of caspase-8 and caspase-3. Caspase-8 as an apoptosis effector, is always involved in extrinsic apoptosis mediated by death receptors [26], but recent literatures show caspase-8 is accompanied by autophagy, and that active form of casapse-8 can degraded by autophagy, which serves as a balance to control autophagy and apoptosis [27–29]. Our results demonstrate that caspase-8 decreases along with the activation of autophagy induced by RSV, but inhibition of autophagy by 3-MA or Bafilomycin A increases the active form of caspase-8, confirming previous reports that autophagy decreases cleavage of caspase-8. Our results also demonstrate that after treatment with RSV, caspase-8 induces the cleavage of Beclin-1, leading to inhibition of autophagy. Our results showing that inhibition of caspase-8 activation prevents Beclin-1 cleavage are in agreement with prior reports, which show that cleavage of Beclin-1 by caspase family members results in inactivation of autophagy [15, 16].

Autophagy and apoptosis both are essential biological processes in maintaining cell stability, structure and function, and proper development. We demonstrate that RSV sequentially induces autophagy and apoptosis, with autophagy as an initial response prior to apoptosis, supporting previous reports [30, 31]. We also show that RSV induces activation of caspase-8 to cleave Beclin-1 and induce apoptosis through the intrinsic mitochondrial pathway. Previously, *Li H et al*. [16] demonstrated that cleavage of Beclin-1 induces the release of cytochrome C, followed by apoptosis after cells undergo the process of autophagy. In addition to Beclin-1 cleavage, ATGs such as ATG5 are also found to be cleaved by caspases in apoptotic cells [32–34].

P62 is an autophagy-related protein, previous studies showed that P62 is degraded through autophagy [20, 35]. Recent reports demonstrated that p62 provided a signal-organizing interface to recruit poly-ubiquitinated caspase-8 and subsequently allow its full activation. When treated with reagents that induced ER stress or proteaosome inhibition, the cells could activate the apoptosis system directly through caspase-8, without the involvement of death receptor signaling [36]. This novel mechanism of caspase-8-mediated apoptosis was dependent on the autophagy-related proteins LC3 and p62 [37, 38]. Thus, in addition to serving as a typical caspase species in the ‘classic’ extrinsic apoptosis, caspase-8 can also be activated in a p62-dependent manner and involved in an alternative endogenous pathway of apoptosis, especially when induced by various reagents or drugs. Our results reveal that P62 decreases along with the activation of autophagy, and increases when autophagy is inhibited. Here we demonstrate that the degradation of p62 triggers formation of Fas/Cav-1 complexes. As far as we know, this is the first report to demonstrate the involvement of P62 in balancing autophagy and apoptosis.

Cav-1, a 22-kDa plasma membrane scaffolding protein, is critical in the formation of the 50- to 100-nm flask-shaped invaginated caveolin [19, 39]. Previous reports demonstrated that Cav-1 regulates caspase 3-mediated apoptotic pathways via down-regulation of Survivin [40]. and the deletion of Cav-1 confers protective functions against both extrinsic and intrinsic apoptosis [41]. The data in our study also confirm that deletion of Cav-1 protects cells from apoptotic death due to an inability to form a death-inducing complex with Fas [42, 43].

In conclusion, we show that activation of autophagy facilitates the degradation of P62, enabling the formation of apoptosis initiating Fas/Cav-1 complexes, followed by cleavage and activation of caspase-8 to trigger apoptosis (Supplementary Fig. 5). Furthermore, caspase-8-regulated cleavage of Beclin-1 also plays a fundamental role in the conversion from autophagic to apoptotic cell death. Although we demonstrate a novel role for P62 in regulating Fas and Cav-1 complex formation, a detailed mechanism is still needs to be elucidated. In particular, p62 has been recently described as a binding partner for many other proteins to facilitate the autophagosomal degradation of ubiquitinated proteins. Further experiments examining the binding sites of P62 and mechanism that regulates the formation of Fas/Cav-1 complexes are warranted.

## MATERIAL AND METHODS

### Chemicals and antibodies

RSV was purchased from Chongqing Kerui Nanhai Pharmaceutical Company, BafA1, DAPI, caspase-3 assay kit, anti-β-actin, LC3, anti-rabbit secondary antibody, anti-mouse- secondary antibody, FITC-labeled mouse-secondary antibody, Cy3-labeled rabbit-secondary antibody were purchased from Sigma (St Louis, MO, USA). RSV was dissolved in DMSO (0.1% v/v final concentration) at 500 mM in stock solution. Antibodies to cleaved caspase-3, Bax, and LC3 were from Cell Signaling Technology (Danvers, MA, USA). Antibodies against Beclin-1 (full-length), P62, Fas, caspase-8, Bcl-2, caspase-3, 3-MA, Z-IETD-FMK, Z-DEVD-FMK were purchased from Santa Cruz (Santa Cruz, CA, USA). Beclin-1 (C-terminal), Cav-1, Atg5, cytochrome-C antibodies were purchased from Biosynthesis Biotechnology (Beijing, China). Dulbecco's modified Eagle's medium (DMEM) and Lipofectamine 2000 were obtained from Invitrogen Biotechnology (Camarillo, USA). Fetal bovine serum was purchased from Excell Biology Co, LTD (Shanghai, China). siRNAs were obtained from Shanghai GenePharma Co., Ltd (Shanghai, China).

### Cell lines and treatments

Human non-small cell lung cancer (A549) cells were purchased from the American Type Culture Collection (ATCC), and cultured in DMEM medium supplemented with 10% (v/v) heat-inactivated fetal bovine serum, 100 U/ml penicillin and 100 U/ml streptomycin at 37°C in a humidified atmosphere with 5% CO_2_. All experiments were performed during the exponential phase of cell growth. After reaching 80% confluence, cells were treated with RSV as indicated and combined with other chemicals.

### Cell proliferation assay

Exponentially growing cells were plated in 96-well culture plates at a density of 7 × 10^3^ cells/well. After plating for 12 h, cells were pre-incubated with corresponding inhibitors for 1 h, then 50 μM RSV was added to the culture medium, the group with no RSV was used as blank control. After incubation for the indicated time, 20 μl MTT (500 μg/ml final concentration) was added to each well, followed by a 4 h incubation at 37°C, then medium was removed and 150 μl DMSO added to each well. Absorbance at 490 nm was read using a Micro-plate Auto-reader (Labsystem). The percentage of viable cells was calculated as follows: cell viability (%) = OD _treatment_ / OD _control_ × 100%.

### Measurement of caspase-3 activity

Caspase-3 activity was determined using a commercially available caspase-3 assay kit (Sigma) as described by the manufacturers. Briefly, after lysis, the Bradford method was used to determine protein concentration. Supernatants were incubated with 2 mM caspase-3 substrate (Ac-DEVD-pNA) at 37°C for 1 h, and then the absorbance at 405 nm was measured with a spectrophotometer. Caspase-3 enzymatic activity in cell lysates was directly proportional to the color reaction.

### siRNA transfection

For siRNA interference, cells were grown to 80% confluence in DMEM growth medium and transfected using Lipofectamine 2000 without serum and antibiotics according to the manufacturer's instructions. The target sequences were 5′-GGAGCCAUUUA UUGAAACUTT-3′ (sense) and 5′-AGUUUCAAUAAAUGGCUCCTT-3′ (antisense) for Beclin1, 5′-GUGACGAGGAAUUGACAAUTT-3′ (sense) and 5′-AUUGUCAAUUCCUCGUCACTT-3′ (antisense) for P62. 5′-CCUUCACUGUGACGAAAUATT-3′ (sense), 5′-UAUUUCGUCACAGUGAAGGTT-3′ (antisense) for Cav-1. After transfection for 24 h, cells were directly used for follow-up experiments.

### Immunofluorescence staining and microscopy

A549 cells were inoculated on coverslips overnight and then incubated with different combined chemicals for the indicated time. Cells were then fixed with 4% formaldehyde at room temperature for 10 minutes. After rinsing with ice-cold phosphate-buffered saline (PBS cells were permeabilized by 0.1% TritonX-100 in PBS for 10 minutes, and then blocked with 10% bovine serum albumin (BSA) for 1 h at room temperature (RT). Coverslips were incubated with 1:500 corresponding primary antibodies overnight at 4°C, followed by CY3-labeled anti-rabbit or FITC-conjugated anti-mouse secondary antibody for 1 h at RT in the dark. Slides were washed repeatedly with PBS, and counterstained with DAPI. Fluorescence was acquired using a fluorescence microscope (Zeiss, Germany).

### Fluorescence intensity analysis

For quantitative analysis of fluorescence intensity, A549 cells were planted in black 96-well culture plates. After cells reached to 80% confluence, cells were treated for the indicated time and dose of RSV, stained with fluorescent dye and then analyzed with a Multiskan Spectrum (Molecular Devices, SpectraMax M2e 200-100) at an excitation wavelength of 507 nm and emission wavelength of 529 nm for Rho123, excitation wavelength 490 nm and emission wavelength 516 nm for MitoTracker Green.

### Co-immunoprecipitation assay

A549 cells were either transfected with siP62 or siControl for 24 h, and then treated with 50 μM RSV for the indicated time. Cells were harvested and lysed in ice-cold RIPA buffer (50 mM Tris–HCl pH 7.4, 150 mM NaCl, 1 mM EDTA, 1% Triton X-100, 1% sodium deoxycholate, 0.1% SDS, 1 mM PMSF, 5 mM aprotinin, leupeptin, pepstatin) for 30 minutes. Total cell extracts were centrifuged at 12,000 g for 15 min at 4°C. To immunoprecipitate endogenous Fas and Cav-1, 1 mg of the total lysate was incubated at 4°C with 2 μg of corresponding antibodies and 50 μl Dynabeads^®^ protein G (Invitrogen, Oslo, Norway). The immune complexes were then washed three times with PBS and followed by lysis buffer. Samples were heated in SDS sample buffer and processed by western blotting.

### Flow cytometric analysis

For assessment of apoptosis, an Annexin V-PI staining kit (Santa Cruz) was used according to the manufacturer's protocol. Briefly, cells were pre-treated with or without inhibitors for 1 hour, followed by treatment with 50 μM RSV for the indicated times. Both floating and adherent cells (removed by trypsinization) were collected. Cells were resuspended in PBS three times, followed by the addition of annexin V-FITC and PI to the binding buffer. Samples were then analyzed by flow cytometry. FITC and PI signals were detected at 518 nm with FL1 and at 620 nm with FL2, respectively. The log fluorescence values of annexin V-FITC and PI are shown on the X and Y axis, respectively.

### Western blotting analysis

A549 cells were incubated with the indicated drugs and times, cells were harvested, washed with PBS and lysed in ice-cold RIPA lysis buffer for 30 minutes. For western blot analysis after protein quantification with a BCA assay kit, 50 μg of denatured protein samples were subjected to SDS-PAGE and probed with the corresponding antibodies as indicated at 4°C overnight, followed by incubation with a 1:10,000 dilution of HRP-conjugated secondary antibody for 1 h at room temperature. The transferred proteins were visualized with a SuperSignal West Dura detection kit (Pierce, Rockford, USA) and exposed to Medical Blue X-ray film. All samples were normalized to β-actin.

### Quantitative reverse-transcription PCR (Q-RT-PCR)

Total RNA was extracted using Trizol reagent (Invitrogen) and then reverse transcribed with a Primpscript® RT reagent kit from Takara Bio Inc. (Tokyo, Japan). Resulting cDNAs were quantified by real-time PCR on a LightScanner 32 Detection System (Idaho, America) using the following primers: Beclin1: 5′-AGGTTGAGAAAGGCGAGACA-3′ (sense) and 5′-GCTTTTGTCCACTGCTCCTC-3′ (antisense); P62: 5′-AGCGTCAGGAAGGTGCCATT-3′ (sense) and 5′-TTCTCAAGCCCCATGTTGCAC-3′ (antisense). Data was normalized to GAPDH transcript levels using 5′- GGCCTCCAAGGAGTAAGACC-3′ (sense) and 5′-AGGGGAGATTCAGTGTGGTG-3′ (antisense) primers. Expression was calculated using the ΔΔC_t_ method.

### Subcellular fractionation

Mitochondrial and cytosolic subcellular fractions were isolated by cell disruption followed by differential centrifugation and washing, as described in the manufacturer's instructions. Briefly, lysis homogenates were centrifuged at 1,000 g for 10 min at 4°C, and supernatants were then centrifuged at 12,000 g for 15 min at 4°C. Pellets contained the enriched mitochondrial fraction, whereas supernatants contained the cytoplasmic/microsomal fraction, equal amounts of proteins were analyzed by western blotting and then probed with the indicated antibodies.

### Statistical analysis

All experiments were repeated at least three times independently. Data are expressed as mean ± S.D (standard deviation) and analyzed with SPSS 13.0 statistical software (SPSS Inc.). The immunofluorescence intensity was analyzed with Image J software (NIH), and the optical density was measured with Quantity One software (Bio-Rad). The difference between each group was analyzed with the Dennett *t*-test and *P* < 0.05 was considered as statistically significant.

## SUPPLEMENTARY FIGURES



## Abbreviations

3-MA: 3-methyladenine
Cav-1: Caveolin-1
RSV: Resveratrol, 3, 4′, 5-trihydroxy- trans-stilbene
DAPI: 4,6-diamidino-2-phenylindole
DMSO: dimethyl sulfoxide
IP: immunoprecipitation
LC3: light chain 3
MMP: mitochondrial membranes potential
MDC: monodansylcadaverine
SQSTM1: sequestosome1
siRNA: Small interfering RNA
Z-DEVD-FMK Z-IETD-FMK: Z-Asp(OMe)-Glu(OMe)-Val-Asp(OMe)-FMK
Z-IETD-FMK: Z-Ile-Glu (OMe)-Thr-Asp(OMe)-FMK
