# Correction: P62 regulates resveratrol-mediated Fas/Cav-1 complex formation and transition from autophagy to apoptosis

**DOI:** 10.18632/oncotarget.27449

**Published:** 2021-11-09

**Authors:** Jun Zhang, Ke Ma, Tingting Qi, Xiaoning Wei, Qing Zhang, Guanwu Li, Jen-Fu Chiu

**Affiliations:** ^1^ Open Laboratory for Tumor Molecular Biology/Department of Biochemistry, The Key Lab of Molecular Biology for High Cancer Incidence Coastal Chaoshan Area, Shantou University Medical College, Shantou, China

**This article has been corrected:** Due to errors in image processing, the HSP70 band in Figure 3A is incorrect. While analyzing the data, the authors accidentally selected the 8-bands western blot image from Figure 2A and inserted them into the HSP70 pane of Figure 3A. The corrected Figure 3A is shown below. The authors declare that these corrections do not change the results or conclusions of this paper.

Original article: Oncotarget. 2015; 6:789–801. 789-801. https://doi.org/10.18632/oncotarget.2733

**Figure 3:**
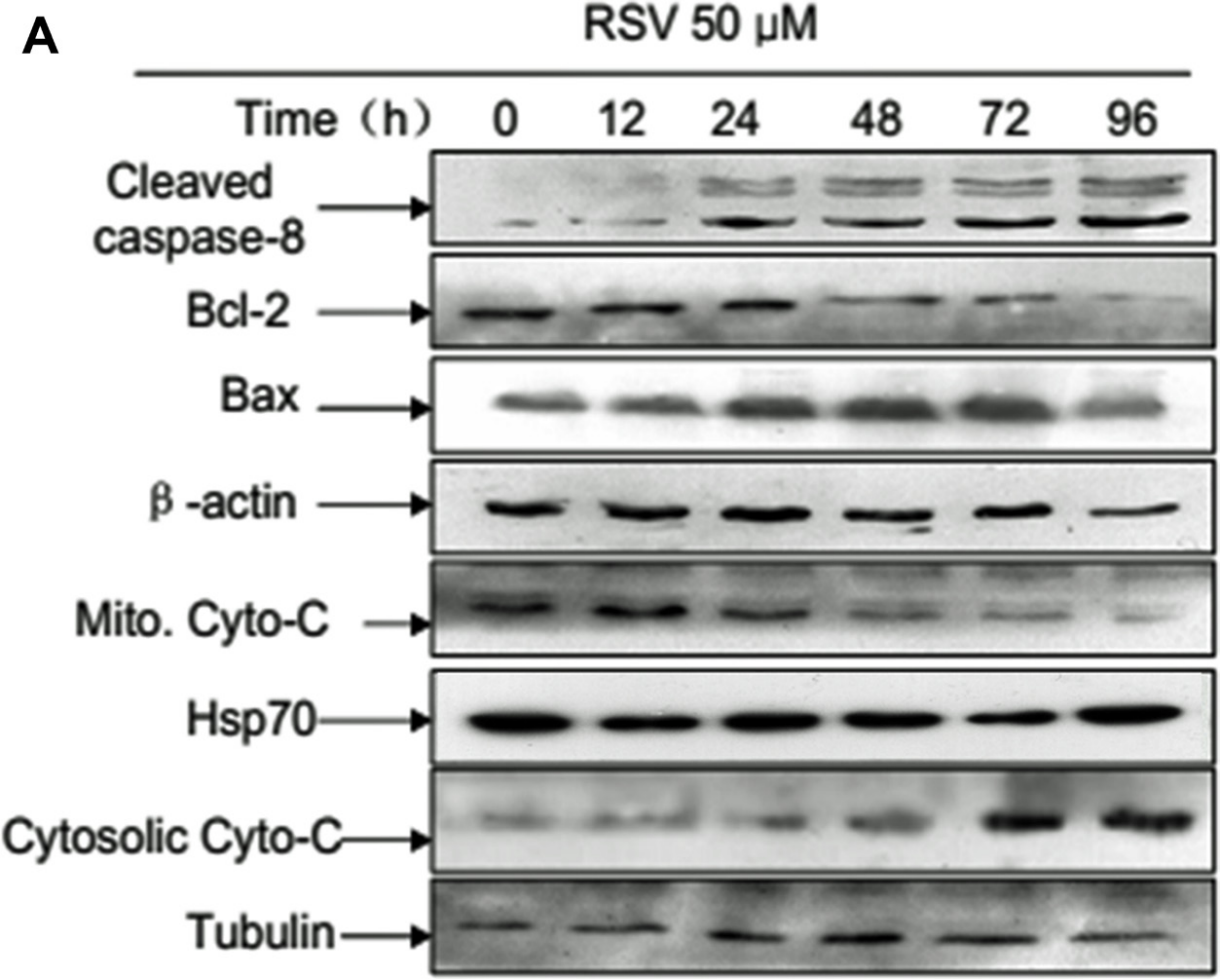
Caspase-8 in autophagy and apoptosis in RSV-treated A549 cells. (**A**) Western blot analysis detected apoptosis-related protein expression levels after treatment with 50 μM RSV at the indicated times. (**B**) Cells were incubated with 50 μM RSV for the indicated times in the presence or absence of Z-IETD-FMK, then cell lysates were used to analyze the expression of cleaved caspase-8, cleaved caspase-3 and LC3 with the specific antibodies. β-actin was used as the loading control. (**C**) Cells were exposed to 50 μM RSV plus Z-IETD-FMK for the indicated times, cell viability was determined by MTT assay. (**D**) Cells were exposed to RSV combined with Z-IETD-FMK for the indicated times. After exposure, cell lysates were obtained and caspase-3 enzymatic activities were measured. (**E**) A549 cells were pre-incubated with or without Z-IETD-FMK and then treated with 50 μM RSV for the indicated times before detection of cell death by flow cytometry after the staining of FITC-conjugated Annexin-V and PI. The histogram represented quantification analysis based on three independent experiments. Data are means ± SD of three individual determinations, *p < 0.05 and **p < 0.01 vs. respective control cells.

