# Supplementary material for: P62 Regulates resveratrol-mediated Fas/Cav-1 complex formation and transition from autophagy to apoptosis
Source: Oncotarget. 2014 Nov 29;6(2):789–801. doi: 10.18632/oncotarget.2733 (PMC4359255; doi:10.18632/oncotarget.2733)
Supplement: Supplementary file 1 [file oncotarget-06-789-s001.pdf]

SUPPLEMENTARY FIGURES

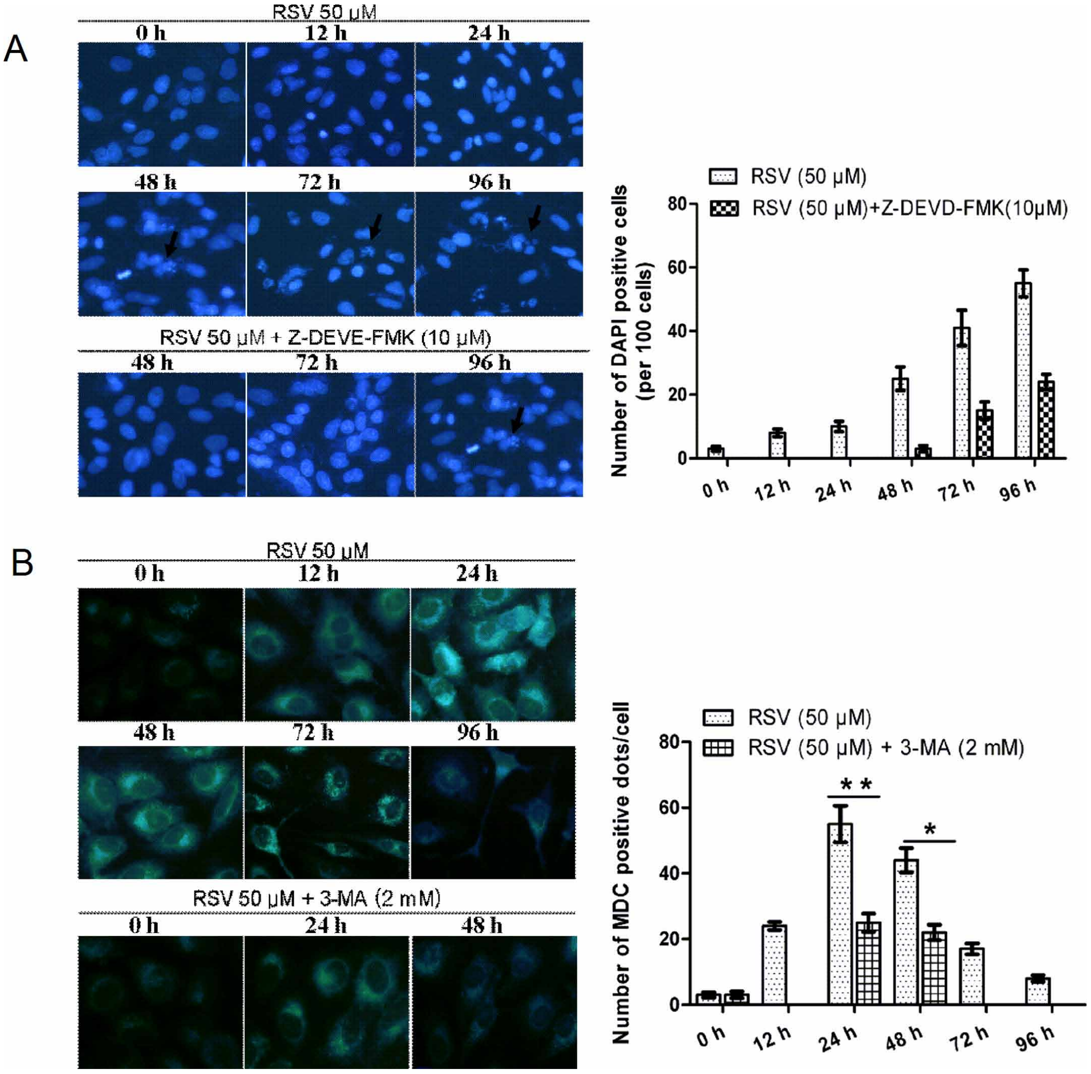

Supplementary Figure 1: RSV-induced autophagy and apoptosis at different time points in A549 cells. Detection of **(A)** nuclear fragmentation and **(B)**, the punctate pattern of autolysosomes, the apoptosis and autophagy related markers in the absence or presence of corresponding inhibitor. Cells were treated for the indicated time with 50 µM RSV, then stained with MDC or DAPI as indicated in the Materials and Methods. Histogram on the right represents the statistical quantitative analysis of autophagic and apoptotic cells. Data are means ± SD of three individual determinations, *$p < 0.05$ and **$p < 0.01$ vs. respective control cells.

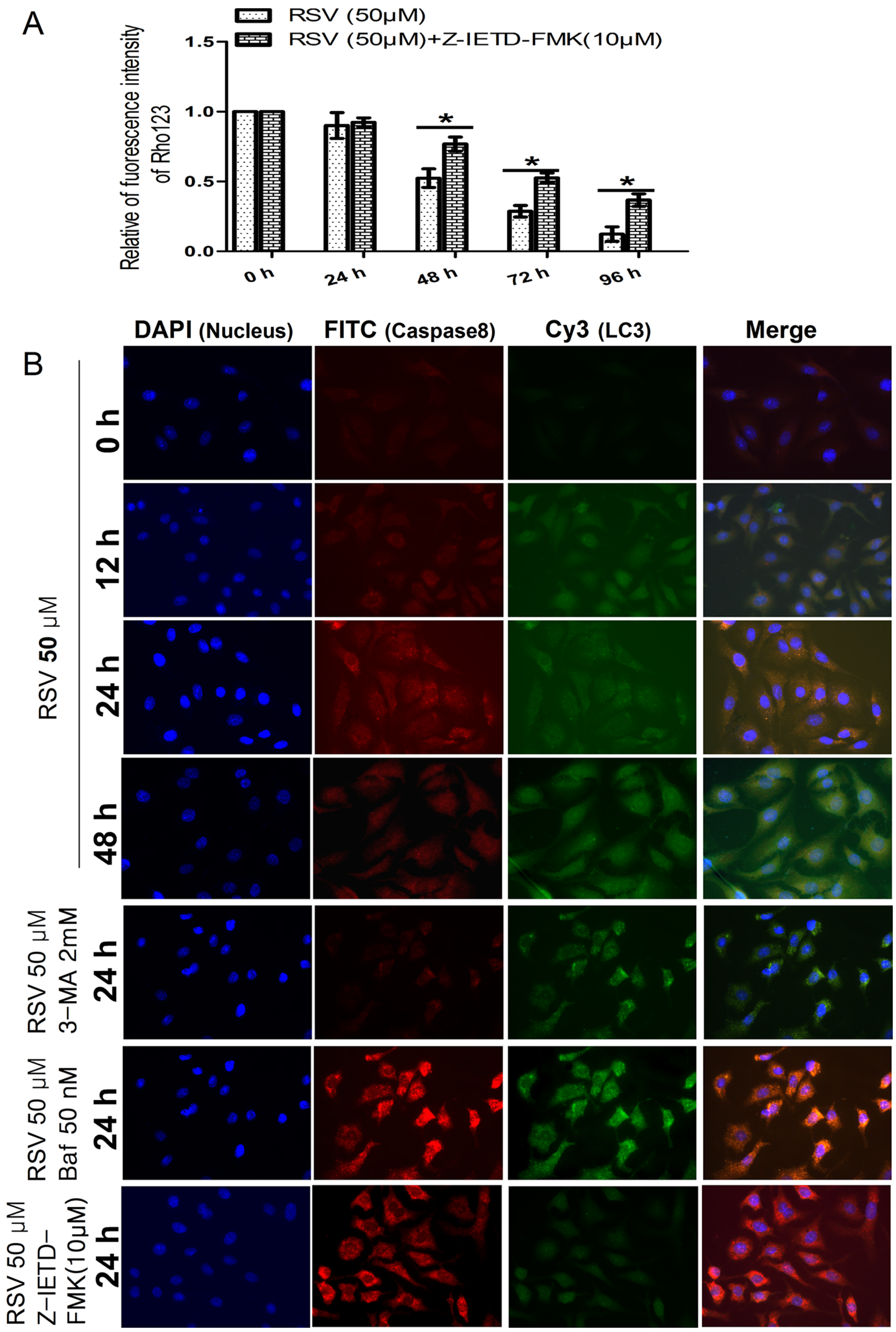

Supplementary Figure 2: Caspase-8 in autophagy and apoptosis in RSV-treated A549 cells. **(A)** MMP was detected after treatment with 50 μM RSV for the indicated times, then cells were stained with Rho123. **(B)** The co-localization between LC3 and cleaved caspase-8 was analyzed by immunofluorescence staining. Cells were stained with anti-LC3, anti-cleaved caspase-8, and DAPI after treatment with 50 μM RSV and inhibitors for the indicated times. The merged images are shown on the right. Data are means ± SD of three individual determinations, *$p < 0.05$ and **$p < 0.01$ vs. respective control cells.

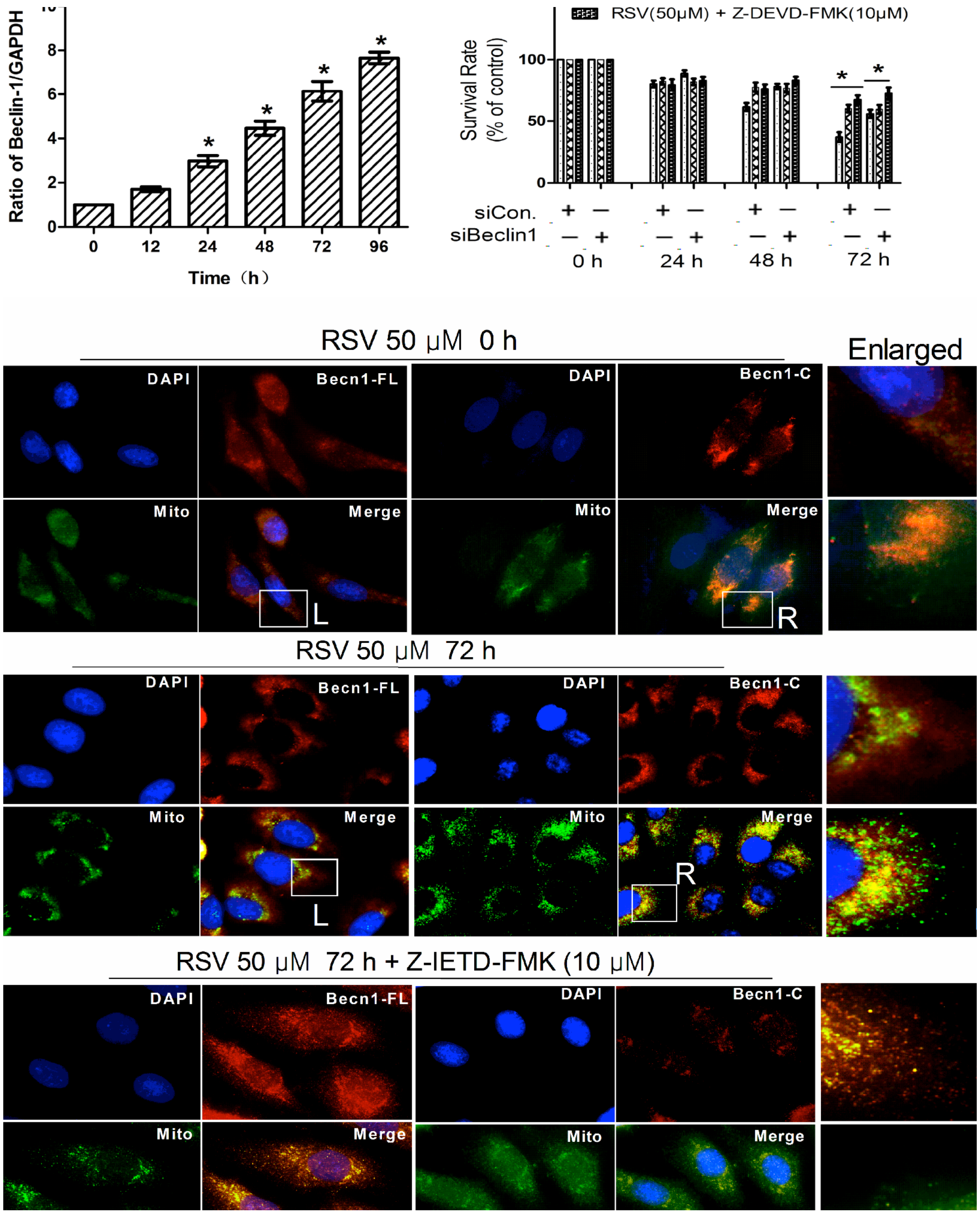

Supplementary Figure 3: Caspase-8 cleaved Beclin-1 inhibits RSV-induced autophagy and promotes apoptosis. (A) Quantitative real-time PCR analyze the expression level of Beclin-1 mRNA were performed after 50 μM RSV treatment for the indicated times. **(B)** The viability of wildtype and Beclin-1 knockdown cells were analyzed by MTT assay. Cells were treated with 50 μM RSV in the presence or absence of caspase-3 and caspase-8 inhibitors respectively. **(C)** The localization of Beclin-1 in mitochondria was analyzed by immunofluorescence staining. After treatment with 50 μM RSV and inhibitors for the indicated times, cells were stained with anti-Beclin-1 (FL), anti-Beclin-1 (C-terminal) and a mitochondrial indicator, MitoTracker. The enlarged part of colocalization area is shown on the right panel (L: Left; R: Right). All data are the mean value of at least 3 independent experiments. *$p < 0.05$ compared with the control cells.

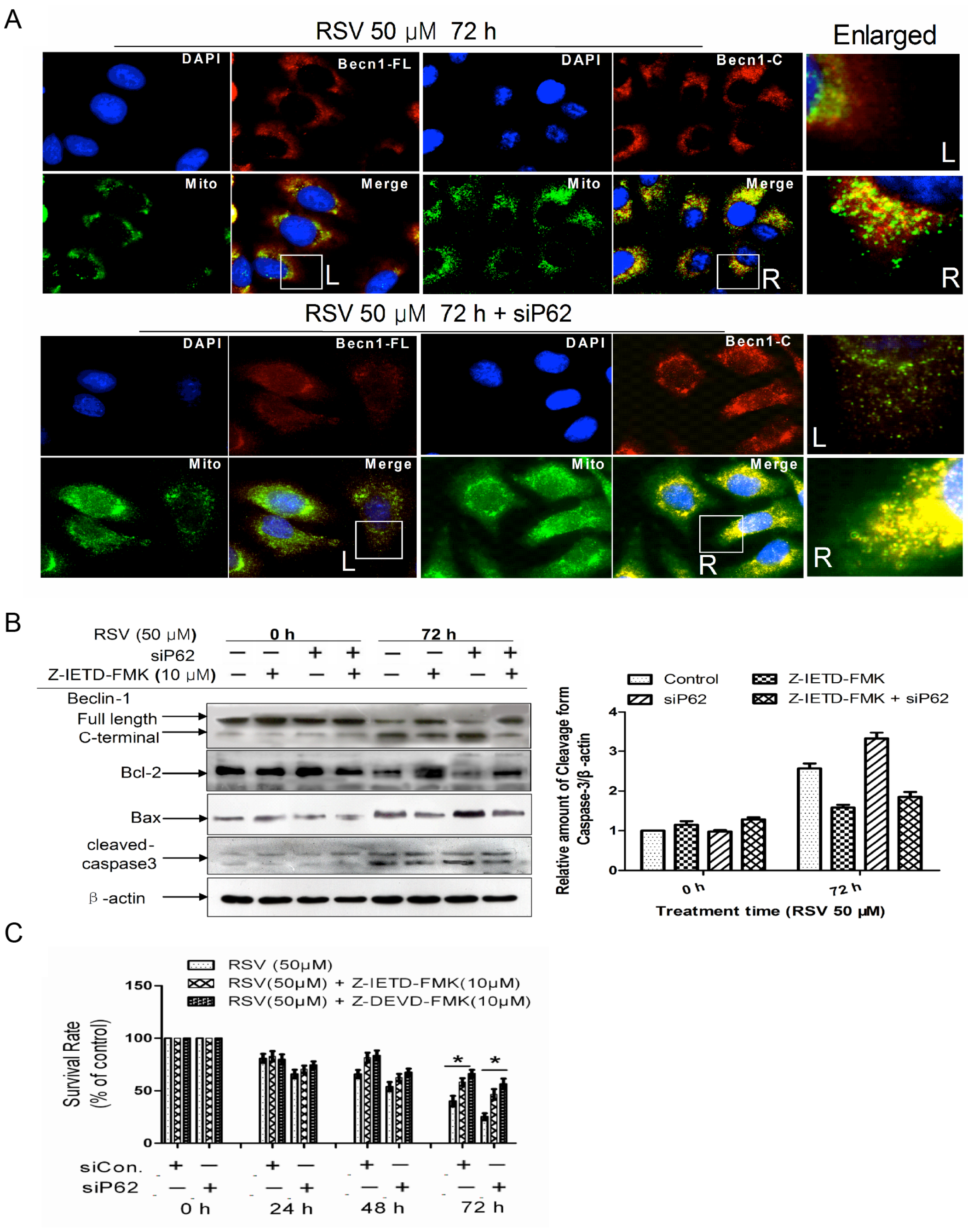

Supplementary Figure 4: Degradation of P62 by autophagy regulates RSV-induced apoptosis through Beclin-1 cleavage. (A) Co-localization of Beclin-1 in mitochondria was analyzed by immunofluorescence staining. Cells were transfected with P62 siRNA, stained with anti-Beclin-1 (FL), anti-Beclin-1 (C-terminal) and a mitochondrial indicator, MitoTrack, and then subjected to microscopy after treatment with 50 µM RSV for the indicated times. Merged images are shown on the right. **(B)** Cells were transfected with P62 siRNA and then treated with 50 µM RSV combined with Z-IETD-FMK for the indicated times, then protein was extracted and analyzed by western blot using the indicated antibodies. The histogram (right) represented quantification analysis based on three independent experiments. **(C)** Viability of wild type and P62 knockdown cells was analyzed by MTT assay. Cells were treated with 50 µM RSV in the presence or absence of caspase-3 and caspase-8 inhibitors, respectively.

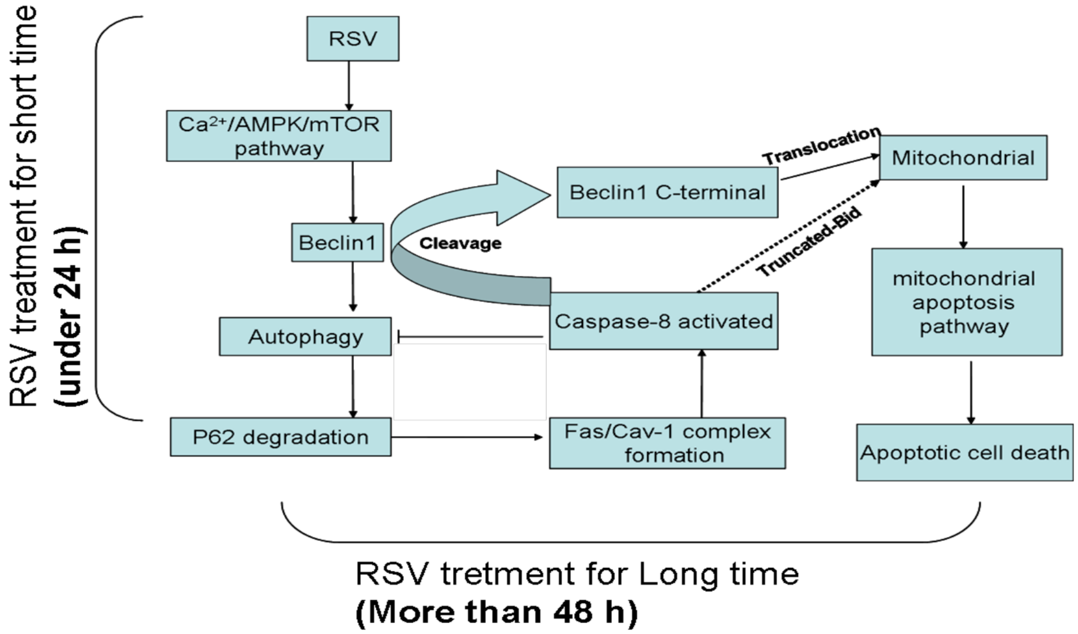

Supplementary Figure 5: Schematic illustration of P62 regulates resveratrol-mediated cell death. Combined with the original data published previously that RSV induced A549 cells to autophagy through the Calcium/AMPK/mTOR pathway [1], here we showed that activation of autophagy by RSV facilitating the degradation of P62, which mediates the formation of the apoptosis initiator Fas/Cav-1 complex, followed by cleavage and activation of caspase-8, which subsequently triggers apoptosis through the intrinsic mitochondrial apoptotic pathway. Furthermore, caspase-8 cleavage fragments function to regulate the cleavage of Beclin-1 whose cleaved products translocate to the mitochondria and mediate the release of mitochondrial cytochrome c to induce intrinsic apoptotic pathways. In addition, the Beclin-1 cleavage products in turn serve as inhibitors of autophagy.

REFERENCE

1. Zhang J, Chiu JF, Zhang HW, Qi TT, Tang QS, Ma K, Lu H, Li GW. Autophagic cell death induced by resveratrol depends on the Ca2+/AMPK/mTOR pathway in A549 cells. Biochem Pharmacol. 2013; 86: 317–328.
